# Hyperpolarized Functional Magnetic Resonance of Murine Skeletal Muscle Enabled by Multiple Tracer-Paradigm Synchronizations

**DOI:** 10.1371/journal.pone.0096399

**Published:** 2014-04-25

**Authors:** Avigdor Leftin, Tangi Roussel, Lucio Frydman

**Affiliations:** Department of Chemical Physics, Weizmann Institute of Science, Rehovot, Israel; University of Michigan, United States of America

## Abstract

Measuring metabolism's time- and space-dependent responses upon stimulation lies at the core of functional magnetic resonance imaging. While focusing on water's sole resonance, further insight could arise from monitoring the temporal responses arising from the metabolites themselves, in what is known as functional magnetic resonance spectroscopy. Performing these measurements in real time, however, is severely challenged by the short functional timescales and low concentrations of natural metabolites. Dissolution dynamic nuclear polarization is an emerging technique that can potentially alleviate this, as it provides a massive sensitivity enhancement allowing one to probe low-concentration tracers and products in a single-scan. Still, conventional implementations of this hyperpolarization approach are not immediately amenable to the repeated acquisitions needed in real-time functional settings. This work proposes a strategy for functional magnetic resonance of hyperpolarized metabolites that bypasses this limitation, and enables the observation of real-time metabolic changes through the synchronization of stimuli-triggered, multiple-bolus injections of the metabolic tracer ^13^C_1_-pyruvate. This new approach is demonstrated with paradigms tailored to reveal *in vivo* thresholds of murine hind-limb skeletal muscle activation, involving the conversion of ^13^C_1_-pyruvate to ^13^C_1_-lactate and ^13^C_1_-alanine. These functional hind-limb studies revealed that graded skeletal muscle stimulation causes commensurate increases in glycolytic metabolism in a frequency- and amplitude-dependent fashion, that can be monitored on the seconds/minutes timescale using dissolution dynamic nuclear polarization. Spectroscopic imaging further allowed the *in vivo* visualization of uptake, transformation and distribution of the tracer and products, in fast-twitch glycolytic and in slow-twitch oxidative muscle fiber groups. While these studies open vistas in time and sensitivity for metabolic functional magnetic resonance studies in muscle, the simplicity of our approach makes this technique amenable to a wide range of functional metabolic tracer studies.

## Introduction

Functional magnetic resonance imaging (fMRI) is an established technology mapping regions of physiological activations and their responses to well-defined stimuli [1]. This mapping is enabled by the changes that physiological processes –and blood oxygenation in particular – bring about in the water's relaxation properties [2], [3]. An alternative, potentially more insightful method to monitor real-time physiology is provided by functional magnetic resonance spectroscopy (fMRS). Instead of focusing on the solvent's signature, fMRS reveals changes in molecular concentrations by monitoring the event-related responses of their spectral peak intensities [4], [5]. Although clearly more closely related to physiological function than water, these metabolite-based observations are considerably challenged by the low concentrations being targeted and by the difficulties involved in reliably and steadily suppressing the H_2_O resonance. Dissolution dynamic nuclear polarization (dDNP) of ^13^C nuclei, is an emerging technology that could provide a potential answer to these challenges [6], [7]. When executed at high fields under cryogenic conditions, DNP can endow stable isotope tracers with upwards of 10^4^ signal enhancements, making up for the low concentration metabolic imbalance. Moreover, ^13^C observations are free from solvent background complications. Dissolution DNP could therefore provide an avenue to monitor real-time metabolic events *in vivo*, with a sensitivity and temporal resolution that are unreachable otherwise.

Limiting this potential are a number of factors, including finite hyperpolarization lifetimes that restrict the *in vivo* observation window to a few minutes at the most, and lengthy sample preparation times requiring hours for hyperpolarizing common tracers such as ^13^C_1_-pyruvic acid [8]. At the conclusion of this process, a dDNP experiment generates a few milliliters of hyperpolarized solution, from which a single bolus with maximal volume defined by the animal being analyzed, is usually administered. For normal mice experiments under conventional infusion conditions, this bolus contains ca. 20–25% of the tracer solution generated by the hyperpolarizer's dissolution process. This administration leads to a “first-pass” kinetic experiment, whose overall duration will be determined by the *in vivo* signal decay time (T_2_) and which can be carried out up to delays given by the hyperpolarized relaxation time (T_1_) [9], [10]. Repeating such hyperpolarized experiment multiple times as needed if attempting to follow stimulation, would require recreating multiple comparable hyperpolarization/reinjection processes –for commonly used substrates this would take several hours, and cease being practical in functional studies.

The present work describes a strategy that enables one to work around this basic problem, by departing from the idea of devoting a full individual DNP dissolution to the monitoring of a single metabolic measurement. Instead use is made of the relatively long T_1_ lifetimes that hyperpolarized tracers can often support at high-fields *in vitro*, to explore the possibilities arising upon injecting the original hyperpolarized solution in multiple boluses associated with independent MRS acquisitions. In particular it is shown that by storing the hyperpolarized solution at high fields and subdividing the total dissolution volume into reduced aliquots, one can collect a series of stimulus-synchronized fMRS and functional magnetic resonance spectroscopic imaging (fMRSI) experiments within an interval bound roughly by the lifetime of the hyperpolarized substrate in the external reservoir where it is kept. This undemanding modification of the usual tracer delivery strategy is broadly applicable to many metabolic studies, and could facilitate new applications in emerging areas including cancer [10], brain [11], cardiac [12], and hepatic [13] research. In the present work, we demonstrate its potential with studies of muscle activation.

Understanding muscle metabolism is an important goal that could benefit from magnetic resonance strategies enabling spectrally and spatially functional investigations of rapidly varying metabolic concentrations. While a large body of research exists on muscle fiber types possessing markedly different phenotypes, molecular-level characterizations of real-time metabolic changes under stimulated *in vivo* conditions remain a challenge. Moreover, because muscle tissue is one of the largest consumers of metabolic fuel, extensive efforts are made to uncover how energetics changes in inherited and acquired diseases such as diabetes [14], muscular dystrophies [15], and myopathies [16]. In view of these challenges and features, and guided by our interest in applying dDNP to monitor stimulated muscle metabolism *in vivo* [17], we chose to focus on the discrimination of different muscle groups to overall physiology upon activation, as a first instance on the potential of dDNP-aided functional MRS and MRSI. To implement such studies a variety of paradigms mimicking muscular exercise were set, and MR measurements were performed in mice in synchrony with both electrical stimuli and with deliveries of hyperpolarized ^13^C_1_-pyruvate into the animals. The *in vivo* conversion of this tracer into lactate was then followed in skeletal muscle. Judging by monitoring this glycolytic response in a purely spectroscopic mode, these studies show that muscle activation involves a graded summation of both intensity- and frequency-driven effects. In combination with spatial localization, these studies showed that stimulation brings about a recruitment of primarily fast-twitch glycolytic muscle fibers, reflecting both transient perfusion and metabolic changes.

## Results

**Figure 1:**
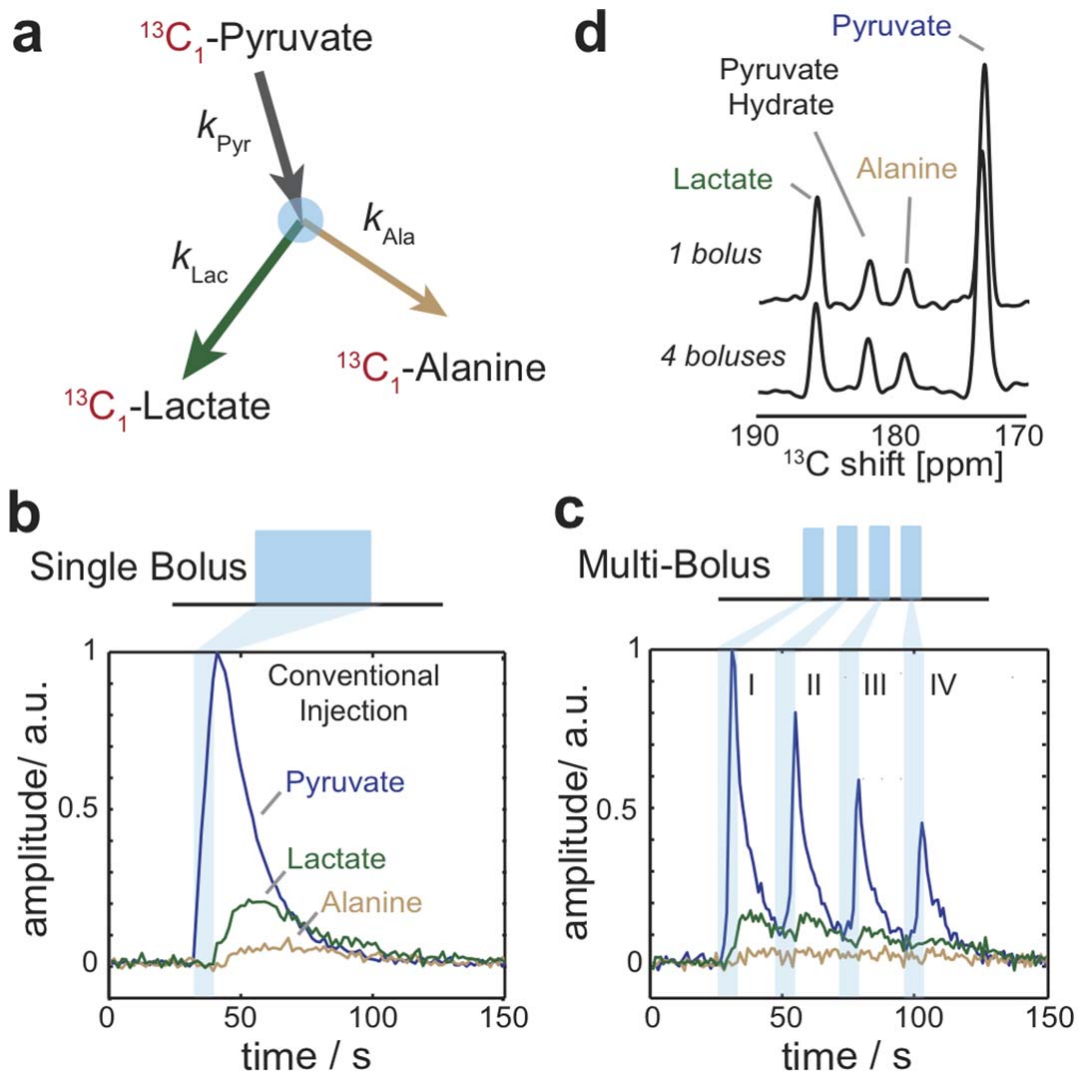
Multiplexing hyperpolarized magnetic resonance using multi-bolus tracer delivery. (**a**) Metabolic scheme showing ^13^C label (red) transfer and respective rate constants for the dynamics of ^13^C-pyruvate, ^13^C-lactate, and ^13^C-alanine. (**b**) Conventional hyperpolarized magnetic resonance spectroscopy of ^13^C-pyruvate metabolism in mouse hind-limb skeletal muscle initiated by a single bolus event (I) with experiment repetition time of 1 s, and pulse flip angle of 15°. (**c**) Experimental demonstration of 4 bolus events (1–IV) with inter-bolus separation of ca. 30 s, experiment repetition time of 1 s, and pulse flip angle of 30°. (**d**) Summed ^13^C spectra from the conventional 1 bolus and multi-bolus measurements showing chemical shifts for observed hyperpolarized metabolites.

Conventional hyperpolarized MRS seeks to monitor metabolism by following the signals that arise from a single injection of a hyperpolarized precursor. During the measurement window initiated by this bolus injection, the observed kinetics will comprise a buildup and decay of the tracer –in the present case ^13^C_1_-pyruvate– followed by subsequent buildups and decays of metabolic by-products such as ^13^C_1_-lactate and ^13^C_1_-alanine (Figure 1a). An example of such hyperpolarized measurement is shown in Figure 1b, for a single bolus injection of hyperpolarized ^13^C_1_-pyruvate into a mouse's tail vein. The detection in this case involved monitoring the bulk ^13^C signals arising from the hind-limb skeletal muscles, using a single-pulse sequence replayed with 1 s repetition times and detecting the ensuing amplitudes with a suitably positioned surface coil. The spectral peaks that can then be resolved exhibit characteristic buildup and decay times, from which metabolic rates can be derived [9], [18], [19]. It has been recently shown that these fluxes characterizing muscle metabolism can exhibit significant changes upon subjecting the animal to a variety of external stimuli [17]. To determine how such external events change the metabolic system as a function of the stimulus duration and/or intensity would require preparing and monitoring a consistent array of hyperpolarized samples for each measurement; with each such process taking approximately 1 hour, applications to monitor real-time changes of *in vivo* metabolism in such settings would be severely limited. Figure 1c illustrates an alternative tracer delivery strategy that allows one to alleviate this limitation. In it a single milliliter-sized hyperpolarized sample has been prepared, and fractions of it (100 µL/2s injection) have been injected into an animal to monitor its metabolism ca. 30 s apart, while keeping the rest of the sample at ∼1 T fields to preserve its nuclear hyperpolarization. Notice how multiple metabolic events can be monitored in such fashion; notice as well (Figure 1d) the good correspondence between a conventional experiment involving a single 400 µL/8 s tracer injection, and the spectral sum arising from the multi-bolus experiment. It follows from this that the number of events that can be monitored in this fashion will be ultimately limited by the duration of the MRS/MRSI acquisition sequence and by the polarization lifetime of the tracer in its high-field reservoir; the volume of initial hyperpolarized solution prepared is usually in excess for mice experiments, and hence of secondary importance in defining this number.

**Figure 2:**
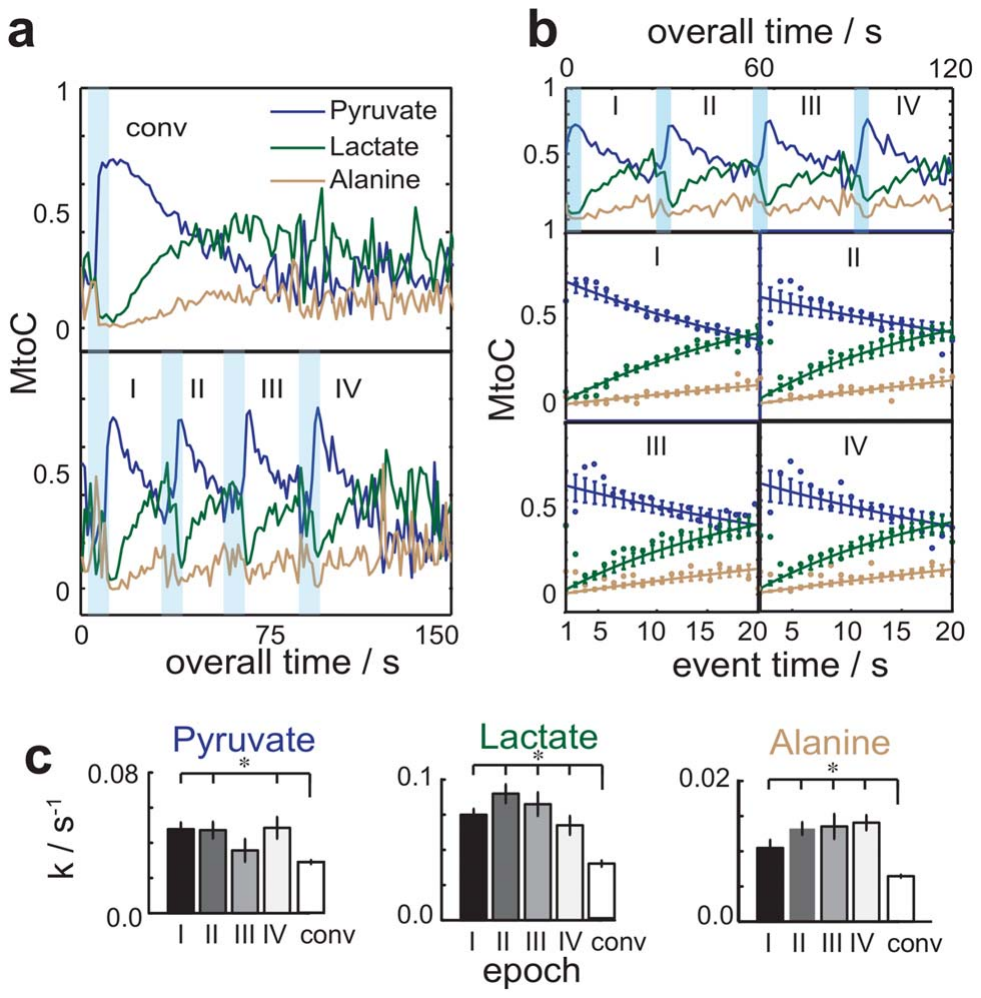
Validation of the hyperpolarized multi-bolus tracer delivery strategy. (**a**) Metabolite-to-total-carbon (MtoC) ratios afforded by: a conventional single bolus (top, 400 µL/8 s) injection experiment, vis-à-vis delivery of four-boluses (bottom, 100 µL/bolus/2s) administered ca. 30 s apart. I–IV denote epochs initiated by the tracer bolus administration, whose duration is illustrated by light blue bands. In all cases a 60 mM hyperpolarized ^13^C_1_-pyruvate solution was injected, following its DNP for one hour. (**b**) MtoC ratios afforded by the multi-bolus functional MRS measurements. (Top) MtoC histories showing the metabolic signal intensity changes for various epochs (blue, green, brown corresponding to Pyr, Lac and Ala respectively). (Bottom) Quantification of metabolic rates *k* from the MtoC histories, showing experimental points and best fits to monoexponential kinetic processes (solid lines). (**c**) Statistical analyses of the rate constants determined in each multi-bolus epoch, compared to the rates arising from a single-bolus conventional injection. Significances were determined by Student's *t*-test, with * indicating *p*≤0.05 for *n* = 4 mice.

In order to validate the metabolic relevance of this technique, a series of multi-bolus tests were conducted under fixed metabolic conditions, and compared against conventional, single-injection benchmark results. These comparisons relied on monitoring the time-dependencies of normalized metabolite-to-total-carbon (MtoC) ratios for hyperpolarized ^13^C_1_-pyruvate, ^13^C_1_-lactate, and ^13^C_1_-alanine, a simple calculation [17] which frees the ensuing results from relaxation-derived effects –both *ex* and *in situ–* at the expense of assuming that these are equal for all metabolites. Figure 2a shows the time-dependent ratios then arising from conventional and multi-bolus injection approaches. The ensuing results can be modeled by a single kinetic event comprising the catabolism of ^13^C_1_-pyruvate and ensuing anabolism of ^13^C_1_-lactate and ^13^C_1_-alanine, each characterized by a single exponential depending on rates *k*_Pyr,_ *k*_Lac_ and *k*_Ala_ (Fig. 1a) [17]; these effective ^13^C_1_-label exchange rates vary in turn, according to the metabolites' pool sizes and the enzymatic activity at the instant of the injection [20]. The multi-bolus MtoC trajectory reveals four sharply delineated events reflecting each of the individual injections; these epochs are labeled as I–IV and quantitatively analyzed for each injection in Figure 2b, whose fits confirm that all injections report on an identical metabolic status. Figure 2c expands this by presenting the exponential rates extracted from repeated multi-bolus and single bolus measurements in hind-limb skeletal muscle experiments on mice (*n = *4). The various multi-bolus rates are statistically identical for each repeated measurement, even if these exceed by approximately two-fold their single-bolus measured counterparts. This systematic deviation probably reflects the fact that upon injecting a larger bolus of pyruvate at supra-physiological concentrations, the hyperpolarized tracer exceeds the metabolic capacity and thereby displays reduced reaction rates [21], [22].

**Figure 3:**
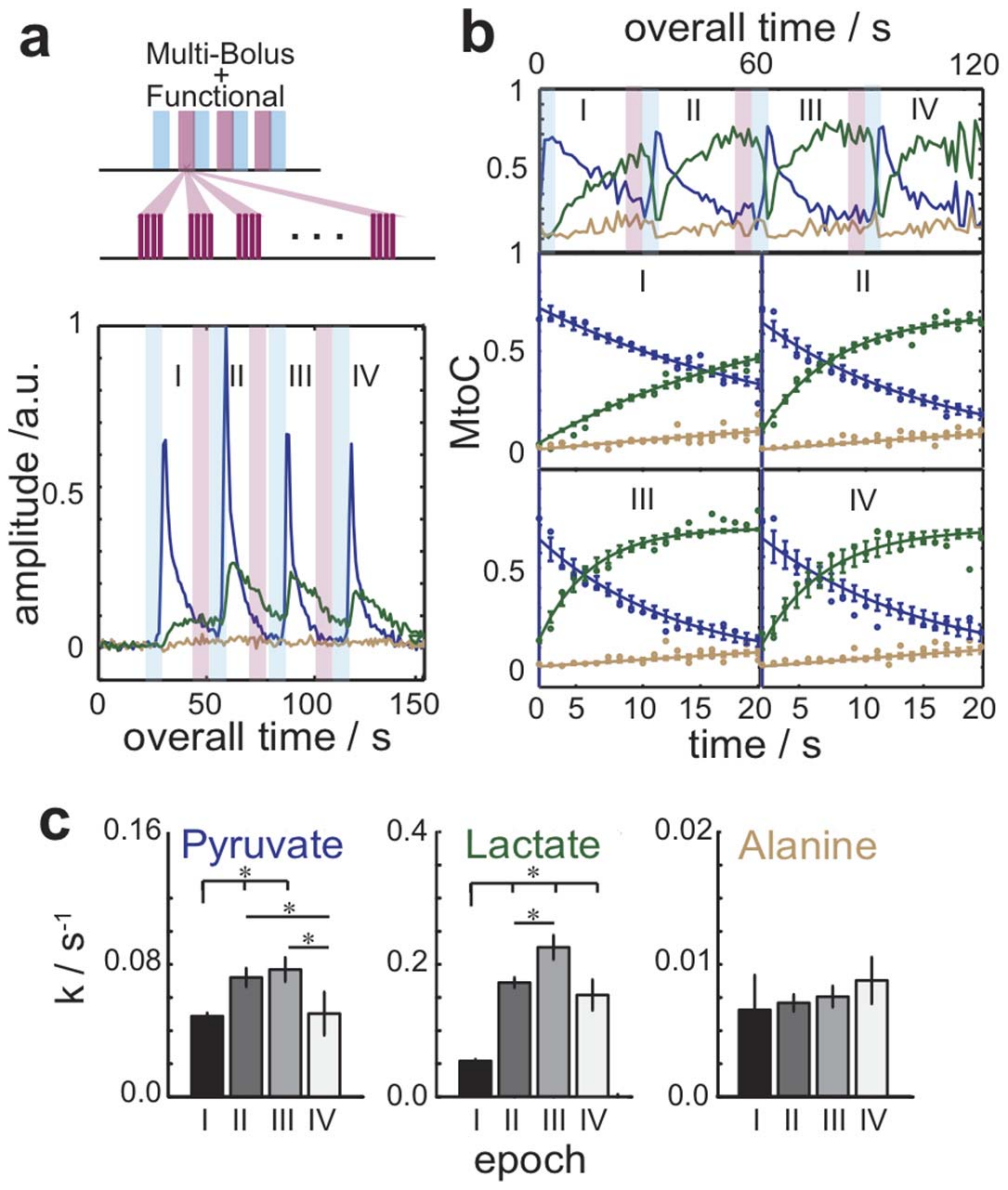
Functional hyperpolarized magnetic resonance spectroscopy using tracer-paradigm synchronization. (**a**) Hyperpolarized multi-bolus fMRS experiment based on an electrical stimulation paradigm. The experiments interleaved the delivery of hyperpolarized pyruvate and surface-coil-based ^13^C MRS acquisitions (blue), with electrical stimulations (red). Four boluses of 100 µL/2s each were delivered with a ca. 30 s separation; stimulations were 10 s long and involved 10 ms trains of 200 µs/10 V pulses repeated at 10 Hz. (**b**) MtoC ratios afforded by the multi-bolus measurements; akin to those shown in Fig. 2b but this time modulated by the presence of stimulation. (Top) MtoC trajectories. (Bottom) Best-fit quantification of each epoch's kinetic data (solid lines). Notice the easily recognizable increase in ^13^C-lactate's production rate in II and III (green curves). (**c**) Statistical analyses of the metabolic rate constants determined for each epoch, with significances determined by Student's *t*-test (* indicates *p*≤0.05 for *n* = 4 animals).

With this corroboration as background, the hyperpolarized multi-bolus MRS experiments were synchronized with a functional model. The activation paradigm chosen mimics the effects of strenuous exercise, by exploiting the response of skeletal muscle to the electrical stimulation of the hind limb sciatic nerve [23]. Figure 3a illustrates the timing and results of these single-pulse/acquire experiments, whereby four 100 µL boluses were injected every ca. 30 s, and a series of ^13^C MR spectra were acquired with a 1 s time resolution. The first of these boluses (epoch I) was administered as control prior to any stimulation; thereafter, each remaining MRS observation was preceded by 10 s periods of electrical stimulation. This resulted in the functional responses labeled as II-IV in Figures 3a, 3b. A first feature evidenced by these functional tests is an increased uptake of hyperpolarized pyruvate, as witnessed by an overall increase in the absolute signals of all metabolites upon activation. Translation of the ^13^C MRS data into MtoC plots also indicates that the ^13^C_1_-lactate ratio increases notably more than their ^13^C_1_-pyruvate and ^13^C_1_-alanine counterparts, confirming the onset of glycolytic activation. Figures 3b and 3c quantify these changes elicited by the functional stimulation; notice how rates in the Epoch I run resemble the non-stimulated controls shown in Figure 2c, while stimulation causes a definite change in the quantified metabolic rate coefficients *k* –evidencing an increase of the label exchange due to the augmented metabolite pools present in the tissue under such stimulus conditions [24]–[27]. Figure 3c in particular reveals the statistically significant *treppe*, or staircase, effects in the metabolic response of the ^13^C_1_-pyruvate and ^13^C_1_-lactate tracer in the hind limb skeletal muscle, from the repeated application of this functional stimulation paradigm. From a physiological standpoint, these steps are hallmark of the early period of stimulus action where substances such as lactate, potassium, and carbon dioxide exert an augmenting affect on tissue activation as their concentration increases, while for extended stimulation periods such substances diminish tissue function [28].

**Figure 4:**
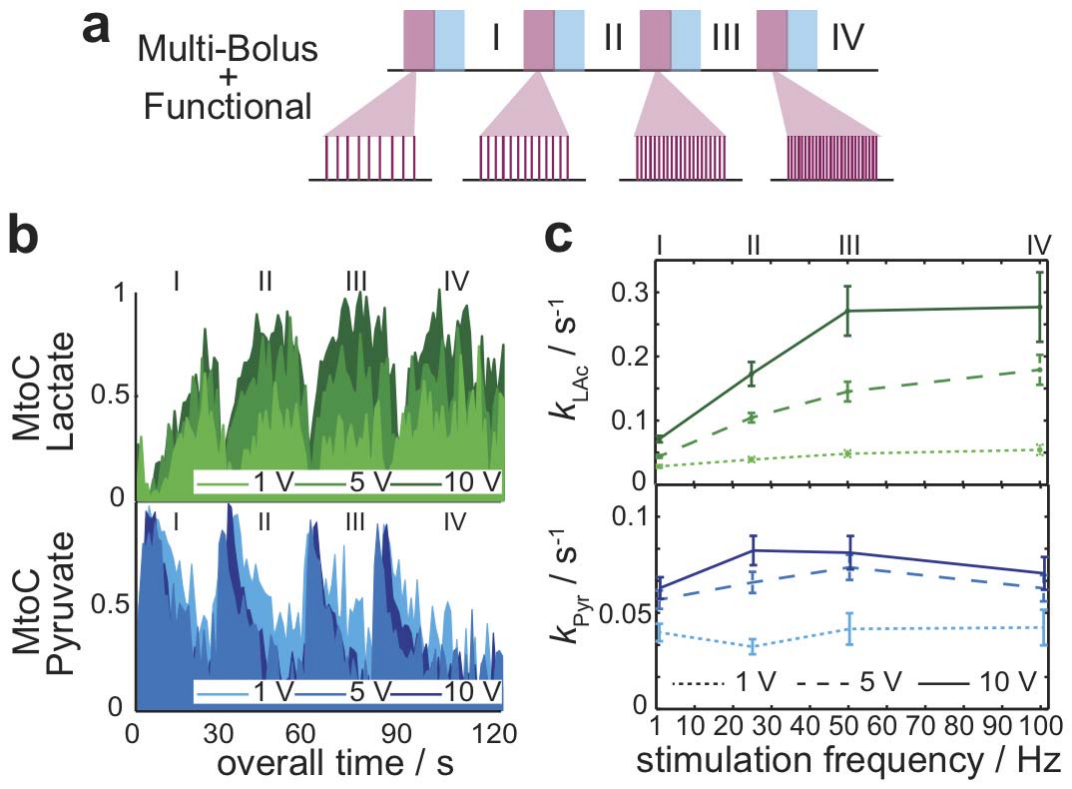
Graded muscular metabolic response monitored with hyperpolarized fMRS. (**a**) Multi-bolus delivery paradigm whereby hyperpolarized ^13^C MRS acquisitions are interleaved with either variable-frequency or variable-amplitude stimulations. (**b**) MtoC trajectories displayed by the injected ^13^C_1_-pyruvate and the resulting ^13^C_1_-lactate monitored as a function of varying frequency and amplitude electrical muscle stimulations. Experiments were carried out as described in Figure 2, with epochs I-IV initiated by injecting 100 µL/2s boluses of 60 mM ^13^C-pyruvate, separated by ca. 30 s of data acquisition. (**c**) Rate constants reflecting pyruvate's disappearance (*k*_Pyr_) and lactate's appearance (*k*_Lac_) as a function of the stimuli's frequency and voltage, arising by fitting the kind of fMRS data summarized in panel (**b**) for *n* = 4 animals.

In order to clarify whether the stimulation-dependent metabolic measurements summarized in Figure 3 exhibit a response that is dictated by a frequency-dependent and/or by an amplitude-dependent recruitment of the muscle fibers responsible for the contraction, the experimental fMRS approach was modified as illustrated in Figure 4a. By contrast to what is shown in Figure 3, this paradigm incorporates now different variable stimuli preceding each epoch. These changes included stimulation blocks that varied either the frequency or the applied voltage of the electrical impulse trains. Metabolite-to-total carbon trajectories arising for representative cases as a function of electrical stimulation are shown in Figure 4b. Analysis of these data leads to the summary in Figure 4c, summarizing the rates of lactate appearance and pyruvate consumption, for *n* = 4 animals and for an array of experiments where the frequencies of the stimulation paradigm were varied between 1 and 100 Hz, and the applied voltages varied between 1 and 10 V. This panel reveals a graded response whereby metabolic fluxes vary in proportion to –but non-linearly with– the activation's amplitude. This response exhibits for all voltages an inflection when the stimuli reach 50 Hz, corresponding to the onset of tetanic impulse summation maximizing muscle fiber contraction [29]. At sub-tetanic frequencies Figure 4c also evidences a response whereby graded metabolism –and by definition, the number of involved motor fibers– increases as a function of the applied voltage.

**Figure 5:**
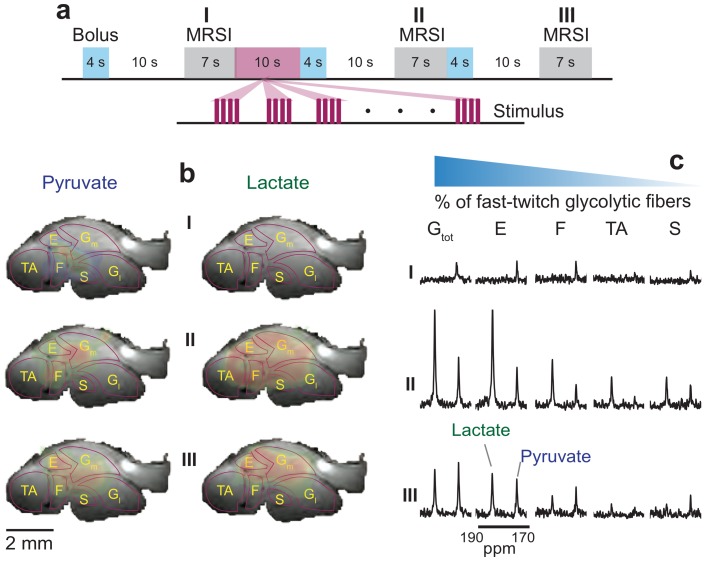
Hyperpolarized fMRSI of muscular activation. (**a**) Multiple-bolus delivery scheme interleaving MRSI acquisitions with off-on-off stimulation paradigms. Control (I), stimulation (II) and post-stimulation (III) epochs were initiated by 133 µL/bolus/4 s injections of 60 mM ^13^C-pyruvate delivered 27 s apart. Stimulation consisted of 10 s strings of 10 V pulse-trains applied with a 10 Hz frequency, and with each train consisting of trains of 200 µs pulses. (**b**) ^13^C quantification maps collected for pyruvate and lactate with a 0.78 mm ×0.78 mm in plane resolution on a 2 mm thick slice, overlaid with anatomical ^1^H MR images obtained with a 0.1 mm isotropic resolution. Indicated in the anatomical reference images are regions assigned to various muscle groups (see text for definitions). (**c**) Summed ^13^C spectra from each region of interest corresponding to the indicated muscle groups (G_tot_, total gastrocnemius), arranged as a function of decreasing percentage of fast-twitch glycolytic fibers.

All fMRS experiments described so far involved non-discriminate spatial observations, whereby disk-like regions ca. 8 mm in diameter and 6 mm in depth were monitored from mice hind limbs via suitable positioning of an external surface coil. Skeletal muscle, however, consists of multiple distinct fiber types that can be discriminated on the basis of their metabolism as fast-twitch-oxidative/glycolytic (FOG), fast-twitch-glycolytic (FG) and slow-twitch-oxidative (SO) fibers [30]. The degree of recruitment of these muscles is determined by the amplitude and frequency of the effort being requested [31], and hence in principle different fibers should be distinguishable by our stimulation paradigm. In order to implement such discrimination, it is necessary to endow the kind of experiments described above with an ability to discriminate the spatial origin of the metabolic responses. To this effect the multi-bolus hyperpolarized paradigm was modified into an MRSI experiment, as shown in Fig. 5a. The experiment involved a first bolus injection (133 µL/4s) followed by a 10 s delay to allow for metabolic evolution [32] and by a 7 s long hyperpolarized ^13^C 2D MRSI acquisition; this was followed by 10 s of functional stimulation and by a second bolus injection (133 µL/4s) and ^13^C MRSI collection; the relaxation of the muscle from an activated to a relaxed state was monitored in epoch III with a final bolus injection (133 µL/4s) and MRSI acquisition. As is customary in both conventional fMRI and hyperpolarized MRSI experiments, these ^13^C acquisitions were complemented with anatomical ^1^H multi-scan MRI images that enabled a clearer identification of the various fiber types, by overlay display on top of the fMRSI ^13^C data. On the basis of these anatomical reference images and of literature comparisons [33], [34], the hyperpolarized ^13^C_1_-pyruvate and ^13^C_1_-lactate resonances were mapped onto major hind-lib muscle groups including the (TA) tibialis anterior, (E) extensor digitorum longus, (F) flexor digitorum longus, (G_m_) medial gastrocnemius, (G_l_) lateral gastrocnemius, and (S) the soleus muscle (Figure 5b). Despite extensive efforts, epoch I experiments revealed only a minor presence of ^13^C_1_-pyruvate and no ^13^C_1_-lactate altogether. This reflects the combined effects of MRSI's reduced overall sensitivity vis-à-vis MRS, coupled to the weak uptake of the hyperpolarized tracer in muscle prior to functional activation (e.g., Fig. 1c). By contrast, the metabolic stimulation and higher rates of transport brought about by the functional paradigm enabled the acquisition of high-resolution maps for both ^13^C_1_-pyruvate and ^13^C_1_-lactate in epoch II. A similar activated post-stimulation metabolic state was also observed in epoch III –despite the fact that no actual stimuli preceded this acquisition. Metabolic map detections in subsequent non-stimulated epochs, acquired using an overall smaller multiple bolus volume reflected reduced sensitivity to smaller bolus volumes, and losses in the polarization of the external tracer in intervals longer than T_1_ of the tracer reservoir.

It follows from these fMRSI maps, that the ^13^C tracer influx is primarily registered with the most glycolytic, outer muscle fibers –in particular the gastrocnemius and extensor muscles. In order to better quantify these spatial metabolic variations, Figure 5c presents the summed ^13^C frequency spectra arising from each of the segmented regions of interest identified in panel 5b. The frequency spectra are arranged from left to right according to the percentage of fast-glycolytic muscle fibers as previously determined in rodents [30], [31]. Here G_tot_ (total gastrocnemius), E and F muscles contain greater than 50% FG fibers and less than 10% SO fibers, TA contains less than 40% FG and is mostly FOG, and S contains approximately no FG fibers and is almost 90% SO [30]. In epoch I only small amounts of ^13^C_1_-pyruvate are detected, predominantly in FG muscles. Epoch II reveals substantial increases in ^13^C-pyruvate and ^13^C-lactate in FG gastrocnemius muscles, yielding 2- and 10-fold integrated intensity increases, respectively, over those observed in epoch I. The soleus exhibited a <2-fold increase in ^13^C_1_-pyruvate upon stimulation, while the ^13^C_1_-lactate signal increased 5-fold. The overall increases of ^13^C_1_-pyruvate in all muscles indicate that stimulation leads to enhanced tracer perfusion, as expected from increases in blood flow previously measured in rodents [35]. The ensuing increase in ^13^C_1_-lactate then reflects both a higher tracer perfusion, as well as an enhanced metabolism. In order to focus on metabolic acceleration and normalize out the effects of T_1_ relaxation over the multiple epochs, the lactate/pyruvate ratios were calculated in pre-stimulation, post-stimulation and refractory periods. Taking the gastrocnemius and soleus as exemplary FG and SO fibers, the pre-stimulus period (epoch I) evidenced noise/pyruvate ratios of ∼0.35 for both muscles. Following 10 s of applied stimulus (epoch II) both FG and SO fibers exhibited similar increases in their pyruvate peaks but distinct changes in their lactates, which rose ∼5- and 3.5-fold over the noise, respectively. This difference in the hyperpolarized lactate signal in FG and SO fibers mirrors the increases in tissue lactate that has been determined *ex vivo* under similar stimulation conditions [25], [26]. The lactate/pyruvate ratio in the refractory period (epoch III), measured approximately 35 s after the end of the stimulus, reveals lactate and pyruvate reductions for both FG and SO muscle fibers –but these remain ca. 150% higher for the former than the latter, vis-à-vis their pre-stimulation baseline values suggesting that FG and SO fibers remain in metabolically stimulated states in proportion to their initial glycolytic activation. Interestingly, in the refractory period, ^13^C-pyruvate signal intensities remain as in epoch II, while 50% reductions in the ^13^C lactate peaks suggest specific metabolic changes. This could be reflecting an oxidation of lactate via the lactate-pyruvate shuttle [36], [37], which will reduce both the intra- and extra-cellular lactate pools generated during exercise. Another possibility is that these fibers are reflecting an early intravasation of muscle lactate, known to occur over longer intervals post-stimulation [38]. However, complementary ^13^C MRS experiments whereby thermally polarized ^13^C_3_-pyruvate was injected under similar stimulus and timing paradigms as in Figure 5's fMRSI experiments, failed to evidence such increases of ^13^C-lactate signals in aliquots of extracted plasma that would result from perfusion-assisted intravasation, in accord with previous measurements under similar stimulation conditions [27].

## Discussion

Hyperpolarized ^13^C tracers can detect *in vivo*, real-time metabolic responses for specific tissues using paradigms and protocols akin to those employed in functional magnetic resonance investigations. This requires modifying the conventional dissolution DNP into a multi-bolus operation; under suitable conditions this can provide unidirectional initial metabolic rates for enzyme-mediated conversions for multiple molecular species, reaction-time dependencies, correlations between metabolism and the intensity of the applied stimulus, and metabolic information discriminated according to position in space. The eventual limit to the amount and the quality of the information that will become amenable by this route, will be given by the number of boluses that can be delivered, by the volume administered in each injection, by the rate of the metabolic process being targeted, by the acquisition strategy used, and ultimately by the lifetime of the hyperpolarized reservoir. In our studies the number of boluses was varied between three and four, but the overall volume administered was kept bound at 400 µL. This is substantially smaller than the overall volume delivered by the hyperpolarizer and it was dictated by physiological considerations; if working with bigger animals, larger boluses could surely be administered. Alternatively, it is possible to use an increasing the number of smaller injections delivered at various intervals while keeping the overall injected volume bound. In unpublished experiments we did in fact successfully use volumes <100 µL, and were able to deliver them in both shorter and longer inter-bolus time intervals in this study. However, signal-to-noise plays an increasingly limiting role at these lower effective concentrations and longer intervals, while short time intervals were not ideal for allowing tracer label exchange to appreciably proceed for our kinetic analysis. Moreover, improvements in experimental efficiency based on more sensitive scanners or reliance on automatic injections [39], could provide needed signal-to-noise for delivering more aliquots possessing smaller volumes and administered over longer durations. Additionally, given the emergence of a new generation of hardware capable of simultaneously polarizing and delivering multiple samples minutes apart [40], hyphenation with a multi-bolus technique could in principle enable literally dozens of real-time measurements collected on a single suitable subject. This would make the method much more akin to conventional fMRI measurements. The tracer reservoir used in our studies was placed in a 1 T field straying from the magnet; under these conditions ^13^C_1_-pyruvate *in vitro* T_1_ exceeds 60 s, enabling us to follow functional variations reliable over the course of 2–3 minutes. Over this duration optimized fast and ultra-fast spectroscopic imaging methods [41]–[43] could prove highly advantageous for implementing in fMRSI modes the kind of kinetic analyses illustrated in Figures 2 and 3 for single-pulse fMRS. Overall, we believe that these hyperpolarized spectroscopy and imaging methods together with multiple-bolus techniques can be realized in widespread applications, including *in vitro* and cellular settings, and *in vivo*; particularly if targeting well-perfused organs such as kidney, liver, and heart, and brain.

In this first demonstration, hyperpolarized multi-bolus functional techniques were applied to examine skeletal muscle activation in mice. Although tracer transport limited the signal-to-noise that even hyperpolarized MR could deliver in these tissues at rest, electrical stimulation enabled first demonstrations of ^13^C fMRS and fMRSI. This led to an increase in signal-to-noise; the rates determined in this manner exhibited a dependence on both stimulation frequency and amplitude, providing a novel perspective into graded muscular responses; it would be hard to envision how the short-time kinetic information provided by hyperpolarization could become available using standard *in vivo* stable isotope tracers or fMRI measurements in exercising muscles. It was also found that metabolic response depended on the type of muscle fiber examined, with primary contributions to the fMRS signal arising from fast-twitch glycolytic muscles supporting rapid anaerobic glycolysis upon stimulation. Fundamentally, the regulation of such metabolic responses are attributed to an enhanced metabolite transport into the tissue thanks to increased blood flow, to the enhanced action of monocarboxylate transporters facilitating the cellular fluxes [44], [45], and to an upregulation of metabolic regulatory pathways [16] that preferentially route the metabolic fuel pyruvate through lactate dehydrogenase reactions under the metabolic challenge presented by our stimulation paradigms [23]. Overall, the multi-bolus assays hereby introduced yield robust kinetic information, in a new real-time kind of measurement monitoring diverse tissue responses *in vivo*. While these protocols were implemented in healthy mice they can be readily extended to discriminate metabolism on muscle disorders. DNP-enhanced fMRS and fMRSI protocols could also have broad potential for studying processes involving a large array of other tissues, and can also be readily extended to *in vitro* and in-cell applications.

## Methods

### Animal Care and Maintenance

Animal protocols and maintenance were in accord with the guidelines of the Committee on Animals of the Weizmann Institute of Science and were approved by this committee (Permit Application Number: 07771212–3). Female 20 week-old ICR mice were maintained in non-reversed light conditions and were provided with standard chow and water *ad libitum* prior to the experiments. Animal respiration was continually monitored and body temperature maintained at 37°C by circulating warm water through the animal holder during magnetic resonance experiments.

### Dynamic Nuclear Polarization

Hyperpolarization of neat ^13^C_1_-pyruvic acid (Sigma Isotec) and 15 mM OX-063 Trityl radical (Oxford Instruments) was conducted at 95 GHz with 50 mW microwave powers in a Hypersense polarizer (Oxford Instruments) operating at 1.4 K. In the multi-bolus experiments the solution was stored at ∼1 T in the fringe field; signal lifetimes measured from the decay of the multi-bolus envelope revealed for this *in vitro* sample a T_1_ = 62.89±6.71 s. *In vivo* hyperpolarized experiments were conducted by injecting 60 mM ^13^C_1_-pyruvate neutralized to pH≈7.6, either in single or multi-bolus volumes as indicated in the text.

### Magnetic Resonance Experiments

All experiments were conducted on a 4.7 T, 30 cm bore scanner (Biospec, Bruker), using an 8 mm ^1^H/^13^C surface coil (Doty Scientific). ^13^C MRS acquisitions were began upon initiating the hyperpolarizer's dissolution sequence. A total of 256 acquisitions (2048 time domain points per acquisition, 4 kHz spectral bandwidths) were acquired in each data set using an inter-scan repetition of 1 s and broadband radio-frequency pulse angles set at 15° in conventional injections and at 30° in the multi-bolus experiments. Each time trace was zero-filled to 4096 points, Fourier transformed, and processed with a 20 Hz Gaussian apodization; kinetics were analyzed using Matlab (Mathworks) routines as described previously [17]. MRSI experiments were conducted using a centric 8×8 phase-encoded matrix spanning a 1.25×1.25 cm^2^ field of view, beginning 10 s after hyperpolarized bolus injections. Ramped-flip angle [46] slice-selective Gaussian pulses were used to obtain free induction decays of 512 time points over 100 ms acquisition times (4 kHz spectral bandwidth); as the acquisition time equaled the phase-encode repetition time, total scan times were 6.7 s. MRSI data sets were reconstructed on Matlab and jMRUI-5 [47] by spatial zero-filling to 16×16 and time domain zero filling to 1024 points followed by 15 Hz Lorentzian apodization. The nominal in-plane resolution of the ^13^C images was 0.78×0.78 mm^2^. Metabolite peaks were quantified using AMARES in the jMRUI-5 software to generate hyperpolarized ^13^C-pyruvate and ^13^C-lactate maps, and quantification error surfaces were used to threshold the maps shown. The metabolic maps are normalized to 5-times the noise level of the acquisition. Reference ^1^H gradient echo images were obtained using TR/TE 5.38 ms/1 s for 1.25×1.25 cm^2^ field, with a 128×128 phase encode matrix.

### Functional Stimulation Protocol

Animals were anesthetized with sodium pentobarbital and electrodes were surgically fastened to the left sciatic nerve of the animal as we described previously [17]. The tail vein was cannulated and the animal fixed in its position inside the magnet and maintained with 1–2% isoflurane in O_2_ at 1 L/min. The electrical stimulation paradigm consisted of variable amplitude (1 V–10 V) 200 μs square pulses played either with a repetition frequency ranging from 1 Hz to 100 Hz, or as 10 ms trains repeated at frequency of 10 Hz.

## Acknowledgments

We are grateful to Prof. Hadassa Degani for valuable insight and discussions, and to Koby Zibzener for assistance with the experiments.

## References

[1] Huettel SA, Song AW, McCarthy G (2009) Functional Magnetic Resonance Imaging (2 ed.). Massachusetts: Sinauer.

[2] Ogawa S, Tank DW, Menon R, Ellerman JM, Kim S-G, et al (1992) Intrinsic signal changes accompanying sensory stimulation: functional brain mapping with magnetic resonance imaging. Proc Natl Acad Sci USA 89: 5951–5955. 1631079 10.1073/pnas.89.13.5951 PMC402116

[3] Ogawa S, Menon RS, Tank DW, Kim S-G, Merkle H, et al (1993) Functional brain mapping by blood oxygenation level-dependent contrast magnetic resonance imaging. A comparison of signal characteristics with a biophysical model. Biophys J 64: 803–812. 8386018 10.1016/S0006-3495(93)81441-3 PMC1262394

[4] Prichard J, Rothman DL, Novotny E, Petroff O, Kuwabara T, et al (1991) Lactate rise detected by ^1^H-NMR in human visual cortex during physiologic stimulation. Proc Natl Acad Sci USA 88: 5829–5831. 2062861 10.1073/pnas.88.13.5829 PMC51971

[5] Mangia S, Tkác I, Gruetter R, Van de Moortele P-F, Maraviglia B, et al (2007) Sustained neuronal activation raises sustained oxidative metabolism to a new steady-state level: evidence from ^1^H NMR spectroscopy in the human visual cortex. J Cereb Blood Flow Metab 27: 1055–1063. 17033694 10.1038/sj.jcbfm.9600401

[6] Ardenkjaer-Larsen JH, Fridlund B, Gram A, Hansson G, Hannsson L, et al (2003) Increase in signal-to-noise ratio of >10,000 times in liquid-state NMR. Proc Natl Acad Sci USA 100: 10158–10163. 12930897 10.1073/pnas.1733835100 PMC193532

[7] Golman K, Zandt R, Thaning M (2006) Real-time metabolic imaging. Proc Natl Acad Sci USA 103: 11270–11275. 16837573 10.1073/pnas.0601319103 PMC1544077

[8] Jóhannesson H, Macholl S, Ardenkjaer-Larsen JH (2009) Dynamic nuclear polarization of [1-^13^C] pyruvic acid at 4.6 tesla. J Magn Reson 197: 167–175. 19162518 10.1016/j.jmr.2008.12.016

[9] Gallagher FA, Kettunen MI, Brindle KM (2009) Biomedical applications of hyperpolarized ^13^C magnetic resonance imaging. Prog Nucl Magn Reson Spectrosc 55: 285–295.

[10] Kurhanewicz J, Vigneron DB, Brindle K, Chekmenev EY, Comment A, et al (2011) Analysis of cancer metabolism by imaging hyperpolarized nuclei: prospects for translation to clinical research. Neoplasia 13: 81–97. 21403835 10.1593/neo.101102 PMC3033588

[11] Mishkovsky M, Comment A, Greutter R (2012) In vivo detection of brain Krebs cycle intermediates by hyperpolarized magnetic resonance. J Cereb Blood Flow Metab 32: 2108–2113. 22990416 10.1038/jcbfm.2012.136 PMC3519415

[12] Schroeder MA, Clarke K, Neubauer S, Tyler DJ (2012) Hyperpolarized magnetic resonance: a novel technique for the in vivo assessment of cardiovascular disease. Circulation 4: 1580–1594. 10.1161/CIRCULATIONAHA.111.024919 PMC3189851 21969318

[13] Lee P, Leong W, Tan T, Lim M, Han W, et al (2013) In vivo hyperpolarized carbon-13 magnetic resonance spectroscopy reveals increased pyruvate carboxylase flux in an insulin-resistant mouse model. Hepatology 57: 515–524. 22911492 10.1002/hep.26028

[14] Petersen KF, Shulman GI (2002) Cellular mechanism of insulin resistance in skeletal muscle. J R Soc Med 95: 8–13. 12216329 PMC1308947

[15] Webster C, Silberstein L, Hays AP, Blau HM (1988) Fast muscle fibers are preferentially affected in Duchenne muscular dystrophy. Cell 52: 503–513. 3342447 10.1016/0092-8674(88)90463-1

[16] Egan B, Zierath JR (2013) Exercise metabolism and the molecular regulation of skeletal muscle adaptation. Cell Metab 17. 10.1016/j.cmet.2012.12.012 23395166

[17] Leftin A, Degani H, Frydman L (2013) In vivo magnetic resonance of hyperpolarized [13C1]pyruvate: metabolic dynamics in stimulated muscle. Am J Physiol Endocrinol Metab 305: 1165–1171. 10.1152/ajpendo.00296.2013 24022866

[18] Hill DK, Orton MR, Mariotti E, Boult JKR, Panek R, et al (2013) Model free approach to kinetic analysis of real-time hyperpolarized ^13^C magnetic resonance spectroscopy data. PLoS One 8: e71996. 24023724 10.1371/journal.pone.0071996 PMC3762840

[19] Kettunen MI, Hu DE, Witney TH, McLaughlin R, Gallagher FA, et al (2010) Magnetization transfer measurements of exchange between hyperpolarized [1-13C]pyruvate and [1-13C]lactate in murine lymphoma. Magn Reson Med 63: 872–880. 20373388 10.1002/mrm.22276

[20] Witney TH, Kettunen MI, Brindle K (2011) Kinetic modeling of hyperpolarized ^13^C label exchange between pyruvate and lactate in tumor cells. J Biol Chem 286: 24572–24580. 21596745 10.1074/jbc.M111.237727 PMC3137032

[21] Hurd RE, Spielman D, Josan S, Yen YF, Pfefferbaum A, et al (2012) Exchange-linked dissolution agents in dissolution-DNP ^13^C metabolic imaging. Magn Reson Med 70: 936–942. 23165935 10.1002/mrm.24544 PMC3660543

[22] Janich MA, Menzel MI, Wiesinger F, Weidl E, Khegai O, et al (2011) Effects of pyruvate dose on in vivo metabolism and quantification of hyperpolarized ^13^C spectra. NMR Biomed 25: 142–151. 21823181 10.1002/nbm.1726

[23] Aslesen R, Engebretsen EML, Franch J, Jensen J (2001) Glucose uptake and metabolic stress in rat muscles stimulated electrically with different protocols. J Appl Physiol 91: 1237–1244. 11509521 10.1152/jappl.2001.91.3.1237

[24] Lindinger MI, Heigenhauser GJ (1988) Ion fluxes during tetanic stimulation in isolated perfused rat hindlimb. Am J Physiol 254: R117–R126. 3337265 10.1152/ajpregu.1988.254.1.R117

[25] Walker PM, Idström J-P, Scherstén T, Bylun-Fellenius A–C (1982) Glucose uptake in relation to metabolic state in perfused rat hind limb at rest and during exercise. Eur J Appl Physiol 48: 163–176. 10.1007/BF00422978 7040072

[26] Lindinger MI, Heigenhauser GJ, Spriet LL (1987) Effects of intense swimming and tetanic electrical stimulation on skeletal muscle ions and metabolites. J Appl Physiol 63. 10.1152/jappl.1987.63.6.2331 3436867

[27] Brechue WF, Stainsby WN (1994) Lactate and acid-base exchange during brief intense contractions of skeletal muscle in situ. J Appl Physiol 77: 223–230. 7961237 10.1152/jappl.1994.77.1.223

[28] Lee FS (1907) The cause of the treppe. Am J Physiol 18: 267–282.

[29] Close R (1964) Dynamic properties of fast and slow skeletal muscles of the rat during development. J Physiol 173: 74–95. 14205033 10.1113/jphysiol.1964.sp007444 PMC1368881

[30] Armstrong RB, Phelps RO (1984) Muscle fiber type composition of the rat hindlimb. Am J Anat 171: 259–272. 6517030 10.1002/aja.1001710303

[31] Armstrong RB, Laughlin MH (1985) Metabolic indicators of fibre recruitment in mammalian muscle during locomotion. J Exp Biol 115: 201–213. 4031765 10.1242/jeb.115.1.201

[32] Yen YF, Kohler SJ, Chen AP, Tropp J, Bok R, et al (2009) Imaging considerations for in vivo ^13^C metabolic mapping using hyperpolarized ^13^C-pyruvate. Magn Reson Med 62: 1–10. 19319902 10.1002/mrm.21987 PMC2782538

[33] Greene EC (1990) The anatomy of the rat. Braintree, MA: Braintree Scientific.

[34] Iwaki T, Hayakawa T (2001) A color atlas of sectional anatomy of the mouse.

[35] Armstrong RB, Laughlin MH (1985) Rat muscle blood flows during high-speed locomotion. J Appl Physiol 1322–1328. 10.1152/jappl.1985.59.4.1322 4055609

[36] Brooks GA (2009) Cell-cell and intracellular lactate shuttles. J Physiol 587: 5591–5600. 19805739 10.1113/jphysiol.2009.178350 PMC2805372

[37] Gladden LB (2004) Lactate metabolism: a new paradigm for the third millenium. J Physiol 558: 5–30. 15131240 10.1113/jphysiol.2003.058701 PMC1664920

[38] Mazzeo RS, Brooks GA, Schoeller DA, Budinger TF (1986) Disposal of blood [1-13C]lactate in humans during rest and exercise. J Appl Physiol 60: 232–241. 3080398 10.1152/jappl.1986.60.1.232

[39] Cheng T, Mishkovsky M, Bastiaansen JAM, Ouari O, Hautle P, et al (2013) Automated transfer and injection of hyperpolarized molecules with polarization measurement prior to in vivo NMR. NMR Biomed 26: 1582–1588. 23893539 10.1002/nbm.2993

[40] Hu S, Larsen P, Vancrieckinge M, Leach AM, Park I, et al (2013) Rapid sequential injections of hyperpolarized [1-^13^C] pyruvate in vivo using a sub-kelvin, multi-sample DNP polarizer. Magn Reson Imaging 31: 490–496. 23107275 10.1016/j.mri.2012.09.002 PMC3625683

[41] Mayer D, Yen Y, Tropp J, Pfefferbaum A, Hurd RE, et al (2009) Application of subsecond spiral chemical shift imaging to real-time multislice metabolic imaging of the rat in vivo after injection of hyperpolarized [1-13C] pyruvate. Magn Reson Med 62: 557–564. 19585607 10.1002/mrm.22041 PMC2782691

[42] Larsen PEZ, Hu S, Lustig M, Kerr AB, Nelson SJ, et al (2011) Fast dynamic 3D MR spectroscopic imaging with compressed sensing and multiband excitation pulses for hyperpolarized ^13^C studies. Magn Reson Med 65: 610–619. 20939089 10.1002/mrm.22650 PMC3021589

[43] Schmidt R, Frydman L (2013) In vivo 3D spatial/1D spectral imaging by spatiotemporal encoding: a new single-shot experimental and processing approach. Magn Reson Med 70: 382–391. 23008051 10.1002/mrm.24470

[44] Coles L, Litt J, Hatta H, Bonen A (2004) Exercise rapidly increases expression of the monocarboxylate transporters MCT1 and MCT4 in rat muscle. J Physiol 561: 253–261. 15388779 10.1113/jphysiol.2004.073478 PMC1665342

[45] Py G, Lambert K, Perez-Martin A, Raynaud E, Préfaut C, et al (2001) Impaired sarcolemmal vesicle lactate uptake and skeletal muscle MCT1 and MCT4 expression in obsese Zucker rats. Am J Physiol Endocrinol Metab 281: E1308–E1315. 11701447 10.1152/ajpendo.2001.281.6.E1308

[46] Xing Y, Reed GD, Pauly JM, Kerr AB, Larsen PEZ (2013) Optimal variable flip angle schemes for dynamic acquisition of exchanging hyperpolarized substrates. J Magn Reson 234: 75–81. 23845910 10.1016/j.jmr.2013.06.003 PMC3765634

[47] Stefan D, Di Cesare F, Andrasescu A, Popa E, Lazariev A, et al (2009) Quantitation of magnetic resonance spectroscopy signals: the jMRUI software package. Meas Sci Technol 20: 10435.

